# Proceedings from an international consensus meeting on ablation in urogenital diseases

**DOI:** 10.1186/s13244-024-01841-2

**Published:** 2024-11-08

**Authors:** Roberto Iezzi, Andrea Contegiacomo, Alessandra De Filippis, Andrew J. Gunn, Thomas Atwell, Timothy Mcclure, Zhang Jing, Alessandro Posa, Anna Rita Scrofani, Alessandro Maresca, David C. Madoff, Shraga Nahum Goldberg, Alexis Kelekis, Dimitri Filippiadis, Evis Sala, Muneeb Ahmed

**Affiliations:** 1 Department of Diagnostic Imaging, Oncologic Radiotherapy and Hematology, A. Gemelli University Hospital Foundation IRCCS, Rome, Italy; 2 Institute of Radiology—Università Cattolica del Sacro Cuore, Rome, Italy; 3 Division of Vascular and Interventional Radiology, University of Alabama at Birmingham, Birmingham, AL USA; 4 Department of Radiology, Mayo Clinic, Rochester, MN USA; 5 Departments of Radiology and Urology, Weill Cornell Medical College, New York, NY USA; 6 Department of Interventional Ultrasound, General Hospital of Chinese PLA, Beijing, China; 7 Section of Interventional Radiology, Department of Radiology and Biomedical Imaging, Yale School of Medicine, New Haven, CT USA; 8 Division of Image-Guided Therapy, Department of Radiology, Hadassah Hebrew University Medical Center, Jerusalem, Israel; 9 2nd Department of Radiology, University General Hospital “ATTIKON”, Medical School, National and Kapodistrian University of Athens, Athens, Greece; 10 Division of Vascular and Interventional Radiology, Beth Israel Deaconess Medical Center, Harvard Medical School, Boston, MA USA

**Keywords:** Interventional radiology, Oncological intervention, Ablation, Urogenital diseases (review)

## Abstract

**Abstract:**

Percutaneous image-guided ablation techniques are a consolidated therapeutic alternative for patients with high preoperative surgical risk for the management of oncological diseases in multiple body districts. Each technique has both pros and cons according to the type of energy delivered, mechanism of action, and site of application. The present article reviews the most recent literature results on ablation techniques applied in the field of genitourinary diseases (kidney, adrenal glands, prostate, and uterus), describing the advantages of the use of each technique and their technical limitations and summarizing the major recommendations from an international consensus meeting.

**Critical relevant statement:**

The article critically evaluates the efficacy and safety of ablation therapies for various genitourinary tract diseases, demonstrating their potential to improve patient outcomes and advance clinical radiology by offering minimally invasive, effective alternatives to traditional surgical treatments.

**Key Points:**

- Ablation therapies are effective alternatives to surgery for renal cell carcinoma.
- Ablation techniques offer effective treatment for intermediate-risk prostate cancer.
- Ablation is a promising tool for adrenal tumor management.
- Ablation reduces fibroid symptoms and volume, offering an alternative to surgery.

**Graphical Abstract:**

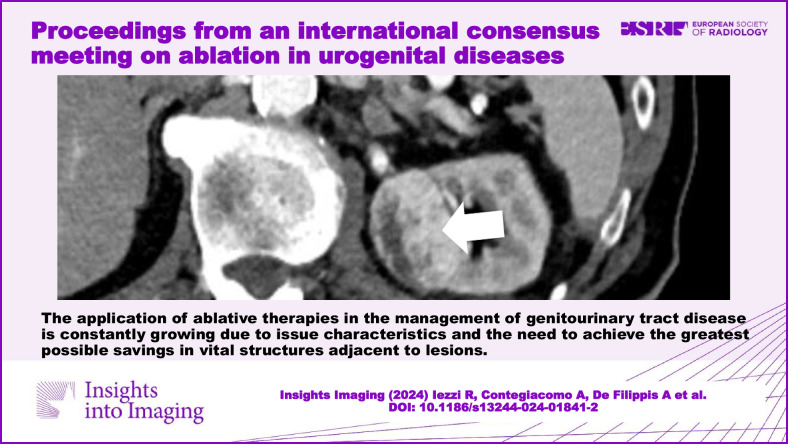

## Introduction

Percutaneous image-guided ablation techniques are growing as valid alternatives to surgery in a wide spectrum of diseases, especially in patients with a high preoperative surgical risk. In the setting of the genitourinary tract, radiofrequency ablation (RFA), microwave ablation (MWA), cryoablation (CA), irreversible electroporation (IRE), and high-intensity focused ultrasound (HIFU) is the most used by interventional radiologists and other specialties [1].

Each one of these techniques has both pros and cons due to the type of energy delivered and the action mechanism. RFA exposes tumor cells to a temperature of 60 °C or higher, with the intent to obtain cellular death. The heat-sink effect is the principal limitation of RFA in lesions proximal to large vessels [2].

The energy transferred by the MWA antenna to the water molecules leads to rapid molecular agitation, frictional heating, and an increase in tissue temperature. Achieving a temperature between 55 °C and 100 °C initiates protein and enzymatic breakdown, and DNA unwinding, resulting in cellular demise and coagulation necrosis. MWA offers various advantages, such as larger ablation volumes, minimal heat sink effect, efficient coagulation of blood vessels, and a swift ablation duration [3].

CA alternates cooling phases, during which an ice ball is produced within the target tissue, and thawing phase. The alternation produces disruption of cell membranes, resulting in cellular cytotoxicity. In addition, CA is less painful and can be performed under conscious sedation [4].

IRE works with high-frequent electric pulses generated between two or more electrodes that produce definitive permeabilization of cellular membranes, causing cell death due to the inability to maintain homeostasis. HIFU uses high-power, highly-focused ultrasound beams to generate heat into a specific tissue. Confined coagulative necrosis is obtained in 1 s treatment due to the high temperature (> 75 °C), with saving of the surrounding tissues [5].

This narrative review article describes the application of ablative techniques in the field of genitourinary tract diseases, exploring the most relevant results of the recent literature and summarizing major recommendations from a consensus international meeting (MIOLive 2023—Rome—ITALY).

## Ablation therapies (AT) for renal cell carcinoma (RCC)

Percutaneous ablation of RCC has been shown to result in oncologic outcomes similar to open nephrectomy [6] and is defining its role as a viable alternative to laparoscopic partial nephrectomy (LPN). The potential role of ablative therapies in reducing hospitalization and complication rates, especially in complex elderly patients, should be compared with the optimal clinical outcomes (radicalization, recurrence, and survival) of surgery which continues to be the standard of care for these patients.

### Ablation alone

In 2015, Ricardo Rivero et al [7] conducted a meta-analysis on 15 studies enrolling about 4000 patients who had undergone CA, RFA, or LPN; in the pooled analysis ablation techniques showed higher general and cancer-specific mortality rates, with equal results with LPN in terms of local recurrence (LR) and distant metastases rates and better complication and postprocedural renal function (eGFR) rates. At the same time, a systematic review by Vollherbst et al [8] on RFA application in RCC management observed that residual tumor (5.9%) and local tumor progression (4.7%) were relatively low and that a tumor size > 3 cm and/or central tumor location are the major risk factors for treatment failure. In the same analysis, RFA showed high success rates in the setting of re-treatment after LR (86.3% for residual unablated tumors and 87.5% for local tumor progression, respectively). Choi et al [9] conducted a 13 articles meta-analysis on MWA showing interesting pooled data on technical success (97.3%) technical efficacy (97.6%), LR (2.1%), major complications (1.8%), and 1-, 2-, 3-, and 5-year cancer-specific survival (CSS) (99.1%, 98.4%, 97.6%, and 96.9%), and overall survival (OS) (98.3%, 94.9%, 86.8%, and 81.9%). In a 2022 meta-analysis by Takafumi Yanagisawa et al [10] on 27 studies AT was shown to be as safe as partial nephrectomy (PN) in terms of renal function preservation and complication rates with a reduced time of hospitalization; in the same paper, cancer mortality and distant metastasis rates were similar between AT and PN with only a slightly lower risk of recurrence in PN patient with cT1b tumors.

A 2023 meta-analysis on MWA on 27 studies with a mixed population of T1a and T1b tumors [11] reported very high pooled success (99.6%) and efficacy (96.2%) rates with very low recurrence rate (3.2%) with a 97.7% CSS rate and 88.1% OS rate at 5 years follow-up, also showing a good minor (10.3%) and major (1%) complication rates. In the same study the sub-analysis of the T1b population (204 patients) showed pooled success and efficacy rates of 100% and 85.2%, respectively, an LR rate of 4.2%, a CSS rate of 98.1% at 5 years, an OS rate of 89.3% at 3 years and a minor and major complication rates of 14.8% and 2.6%, respectively.

Aboumarzouk et al [12] focused their attention on the comparison of LPN and CA in the management of RCC. Their meta-analysis concluded that LPN was more radical than ablation with a higher CSS but with longer hospitalization, worse complications, and higher costs.

A comparative meta-analysis by Timothy McClure [13] on MWA and CA showed that these techniques are equally effective in terms of technical success, OS, disease-free survival, and complications rates but MWA showed a significantly lower mean procedural time and a lower 1-year LR rate. However, the authors concluded that there was a lack of direct comparative studies.

The group of Lin Dong [14] analyzing the results of robot-assisted PN and LPN in comparison with ablative therapies (RFA, CA, MWA, IRE, and SBRT) in a meta-analysis of 26 articles observed better outcomes of ablative therapies in terms of operating time (OP), estimated blood loss (EBL), hospitalization, eGFR, minor and overall complications, with comparable results on LR, distant metastasis, and major complications.

### Combined treatment (embolization and ablation)

**Fig. 1:**
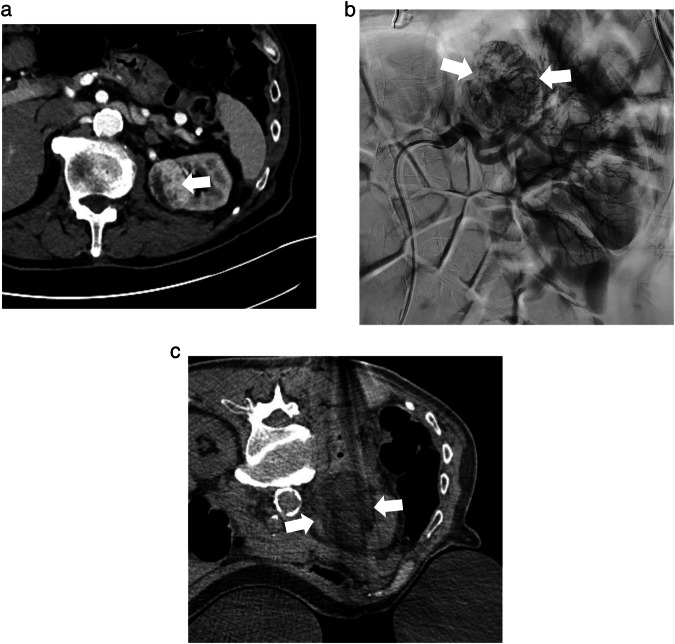
Eighty-three-year-old man with a 4.2 cm biopsy-proven clear cell RCC in the left kidney on contrast-enhanced CT (**a**, white arrow). DSA of the left renal artery shows the hyper-vascular mass in the upper pole of the left kidney (**b**, white arrows). Axial CT obtained during percutaneous CA the following day shows the hypodense iceball surrounding the mass in the left kidney (**c**, white arrow)

Regarding the combination of trans-arterial embolization (TAE) as an adjuvant therapy before ablation, a retrospective comparative study from the Mayo Clinic in the early 2000s [15] including patients with tumors greater than 5 cm in size undergoing pre-procedural embolization, a lower loss of blood during the ablation procedure was observed. Another study by Andrew Gunn et al [16] compared 13 patients who underwent trans-radial embolization before the ablation procedure with 35 patients in which only ablation was performed and an 8.3% rate of major hemorrhagic complications in the ablation group compared to no hemorrhagic complications in the combined procedure group was observed (Fig. 1). Yutaka Nakasone [17] included twelve patients in a study on the combined treatment of RCC with TAE and RFA reporting no major complications, no renal function failure and no instances of recurrence during a mean follow-up period of 47 ± 3.8 months. In a slightly larger series by Duan et al [18] on 28 patients with large tumors (diameter range 4.1–9.6 cm) not candidates for surgery, disease control was achieved in 92.8% of patients in the absence of creatinine levels or urea nitrogen concentrations changes and major complications.

Hideo Gobara et al [19] reported the results of a prospective study on TAE performed using a mixture of absolute ethanol and iodized oil before CA in patients with RCC of > 3 cm. In their population (19 patients), local tumor progression occurred in two patients and no patient had distant metastasis with OS, progression-free survival, and cause-specific survival registered of 95%, 84%, and 100% at 5 years, respectively.

Sommer et al [20] finally conducted a systematic review of the literature concluding that TAE is feasible, safe, and very effective for the treatment of both T1a tumors in challenging locations and T1b tumors to achieve a wider treatment coverage.

## Ablation in adrenal disease

**Fig. 2:**
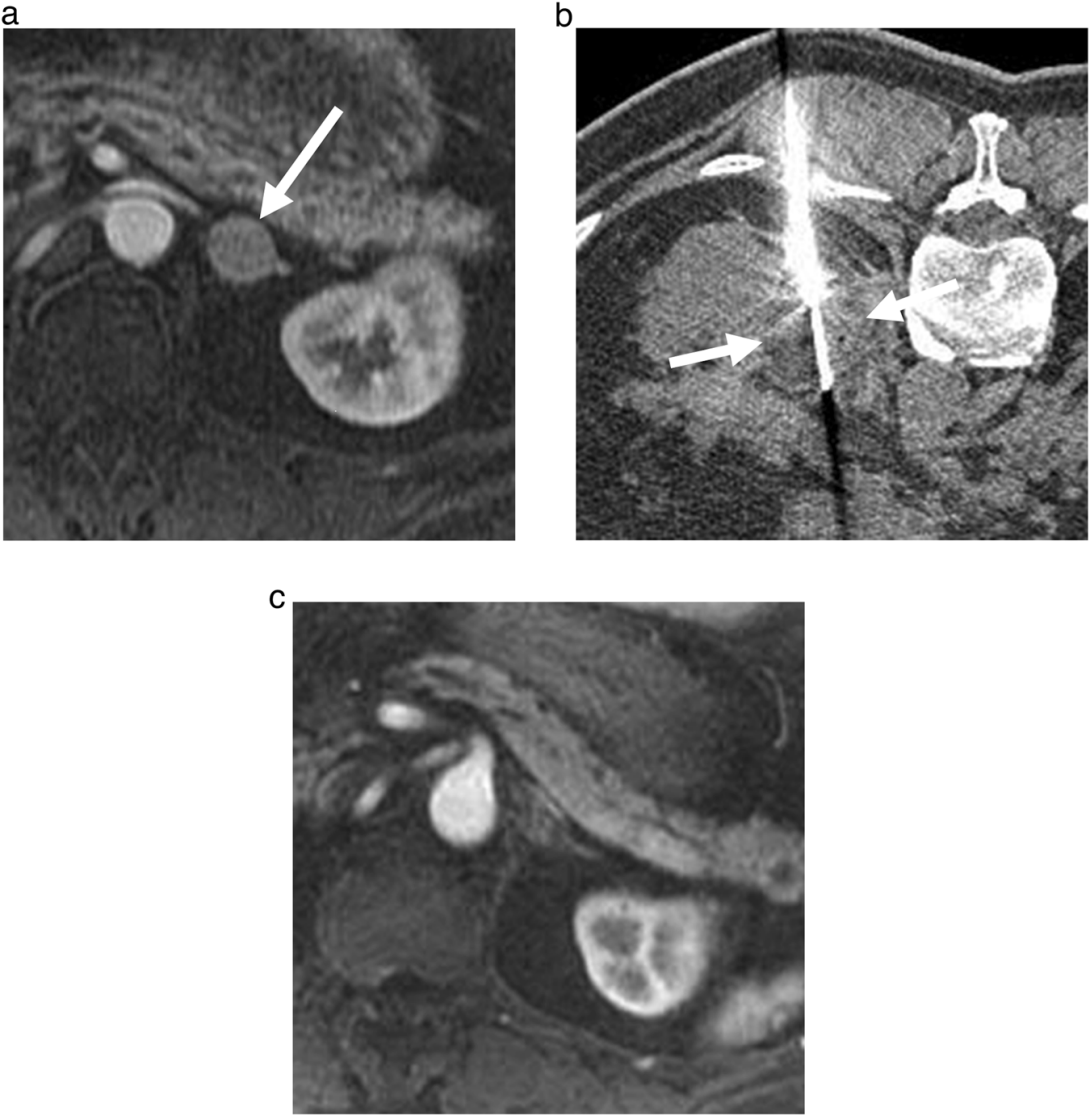
**a** Contrast-enhanced T1 weighted axial MRI image shows a 2 cm left adrenal metastasis from hepatocellular carcinoma (arrow); **b** CT image obtained during CA of the left adrenal metastasis with the patient in the prone position, with arrows showing iceball margin; and (**c**) contrast-enhanced T1 weighted axial MRI image 4 years following CA shows no residual or recurrent tumor

Adrenal ablation can be applied in the management of both malignant and benign diseases. Adrenal tumors comprise a broad spectrum of neoplasms, including primary adrenal tumors (non-functional adenomas, cortisol-producing adenomas, pheochromocytomas, and adrenocortical carcinomas) and metastases. The adrenal gland is a common site of metastatic diseases, representing the most common malignant tumor found in the adrenal gland [21]. Therefore, the first target of adrenal ablation is the management of adrenal metastases (AMs) (Fig. 2).

Following the publication of multiple small case series, a 2021 meta-analysis included 37 studies published between 2009 and 2020, comprising 959 patients with a median tumor size of 3.5 cm [22]. Of the 959 included patients, 33.3% underwent RFA, 7.5% MWA, 9.9% CA, and 4.8% ethanol injections for the treatment of AMS. The remaining 44.4% of patients were from studies involving a mixture of the four listed percutaneous ablation techniques. The OS and local control rates at one year were 77% and 80%, respectively, with similar local control rates for RFA, MWA, and CA. Local control rates were shown to correlate with tumor size. The overall rate of severe adverse events after ablation (CTCAE grade 3 or higher) was 16.1%. Intraoperative hypertension (defined as an acute increase in systolic blood pressure > 180 mm Hg and/or diastolic blood pressure > 110 mm Hg) was the most common adverse event after ablation treatment, occurring in 21% of patients; the majority of which were effectively managed with antihypertensive medications. Less frequently were reported postoperative pain, adrenal insufficiency, pneumothorax, hemorrhage, myocardial infarction or troponinemia, hematuria, and arrhythmia.

In 2022, Zhang et al conducted a similar meta-analysis on 15 studies that enrolled 538 patients with 562 AMs who underwent ablation. The results showed that percutaneous ablation was a safe approach in treating patients with AMs, with low incidence of local hemorrhage, pneumothorax, and hypertensive crisis rates (3%, 6%, and 6%, respectively). The ablation treatment was associated with reasonable local control and efficacy with an 81% local control rate and 1-year OS of 80%, respectively [23].

Cushing syndrome is often caused by the excessive production of adrenocorticotropic hormone (ACTH), either primarily from the pituitary gland or occasionally from an extra pituitary tumor. This results in stimulation of the adrenal glands and hypersecretion of glucocorticoids. Primary treatments are directed at managing the production of ACTH. However, this is occasionally unsuccessful. Limited published experience has shown that the thermal ablation of the hyperfunctioning adrenal glands can be used to treat Cushing syndrome [24].

Several small series have addressed the role of ablation in managing functional adrenal adenomas, as summarized in a meta-analysis published in 2019 by Liang et al [25]. A total of 89 patients from seven studies were included in this analysis. The results revealed a statistically significant decrease in systolic blood pressure (−29.06 mm Hg; 95% confidence interval [CI]), diastolic blood pressure (−16.03 mm Hg; 95% CI), and the number of antihypertensive medications used after ablation. The authors concluded that ablation for aldosterone-producing adrenal adenoma can effectively control blood pressure, reduce the need for antihypertensive drugs, and normalize hormone secretion.

A hypertensive crisis is the most frequent adverse event associated with adrenal ablation [26]. Such hypertension is the result of the cytolytic release of catecholamines from chromaffin cells into the bloodstream during thermal treatment [27]. In an excellent review of 57 patients with AMs treated with ablation, Fintelmann et al showed an association between hypertensive crisis and smaller tumor diameter (< 4.5 cm) and visualization of normal adrenal tissue on CT [26]. While catecholamine release and secondary hypertension are typically observed during active heating with RFA and MWA, such hypertension typically occurs during the thawing phase of CA, presumably related to the slow release of catecholamines from lysed cells [28].

Investigators have found that alpha blockade prior to ablation helps prevent hypertensive crises and decreases the maximum blood pressure compared to the untreated cohort. Such premedication can have drawbacks, as this same study showed that those pretreated with alpha-blockers were more likely to require vasopressors during ablation [29].

## Ablation in prostate cancer management

Prostate cancer management is based on risk stratification using PSA and histology as determinants [30]. Patients with very low or low risk of prostate cancer receive active surveillance repeating PSAs, MRIs, and prostate biopsies. Patients with intermediate-risk or high-risk disease confined to the gland receive radical treatment with either surgical prostatectomy or whole gland radiation—the standard of care, but associated with complications such as urinary incontinence, erectile dysfunction, and negative quality of life impacts, which are of increasing concern to patients [31].

Ablative therapies find their role in the setting of intermediate-risk patients of which the goals are the downstaging of the risk and the prevention of side effects. HIFU, CA, and IRE are the most applied ablative techniques in the field of prostate cancer management.

### Primary prostate cancer (PPC)

Many authors focused their attention on the management of PPC mostly focusing on oncological and functional outcomes. Chen [32] recently published a 10-year experience on 191 intermediate-risk patients reporting biochemical recurrence-free rates of 92.6%, 76.6%, 66.7%, and 50.8% at 1 year, 3 years, 5 years, and 10 years, respectively, with 10 years metastasis-free, cancer-specific, and OS rates of 89.5%, 97.4%, and 90.5%, respectively. The results obtained by Chen on biochemical recurrence were in line with those of Selvaggio et al [33] and slightly higher compared to the large series by Anwar Khan [34], but in this last study, the population also included high-risk patients (14.4%). In the study of Selvaggio [33] complications were analyzed and observed in almost 5% of their population. This result matched with the large series of Wei Phin Tan [35] and the series by Valerio [36], prospectively enrolling 18 intermediate/high-risk patients, describing only one major complication (hematuria), a marked reduction of prostate-specific antigen (PSA), and no signs of disease progression at 1 year MRI follow-up. Another perspective series by Fernández-Pascual et al [37] was larger (75 patients) and focused only on LR which occurred in 15.2% of patients.

HIFU results are comparable to those of CA for PPC. In a prospective study by Duwe et al [38] on 29 patients with PPC treated with HIFU, 44.8% of patients had the persistent disease at 1-year follow-up with relatively low adverse events (70% of patients with sufficient erectile function and 97% with full urinary continence). In the prospective large series by Niklas Westhoff et al [39] half of the patients had complete cancer response at 1-year follow-up with metastasis-free survival and OS of 100% and 98%, respectively. OS was comparable also in the retrospective series by Lo Verde [40], on 97 patients enrolled OS, CSS, and biochemical recurrence-free survival (BRFS) rates resulted to be 91.8%, 100%, and 40.3% on a 10-year observation time, respectively.

Also, IRE finds applications for the management of PCC management. In a recent trial on the oncological outcome of IRE, Zhang et al [41] randomized patients with low- or intermediate-risk prostate cancer to receive either focal or extended IRE ablation, concluding that focal and extended IRE ablation achieved similar oncological outcomes and should be considered for the management of intermediate-risk patients in which surveillance could fail. In the large series by Scheltema [42] 229 patients with low- to high-risk prostate cancer were analyzed in a median follow-up period of 60; among them, IRE obtained failure-free survival rates of 91%, 84%, and 69% at 3 years, 5 years, and 8 years, respectively, with metastasis-free and OS rates 99.6% and 100%, and an acceptable level of side effects.

Finally, a recent meta-analysis by Guo [43] on a large series of studies reported values of pooled BRFS for CA and HIFU of 75.7% and 74.4%, respectively, a CSS for CA, HIFU, and IRE of 96.1%, 98.2%, and 97.9%, respectively, and an OS of 92.8% for CA, of 95.2% for HIFU, and for IRE was 99.5%.

### Recurrent prostate cancer

In the setting of recurrent prostate cancer, Ramalingam et al [44] published a small series on 18 patients with a mean follow-up period of 10.0 months, obtaining a significant reduction of PSA values and a complete response rate of 88.9% with only four patients experiencing minor adverse events. Wimper et al [45] analyzed the results of 114 patients treated with MRI-guided CA including 15 with- and 99 without radical prostatectomy. Outcomes obtained were an overall 1-year and 5-year recurrence-free survival of 76.0% and 25.1% and an overall treatment-free survival of 91.5% and 58.2% at the same time points, respectively, with significantly better oncological results for patients with radical prostatectomy which however experienced a higher rate of complications, predominantly minor. Wei Phin Tan [46] focused on both the oncological and functional outcomes of 110 patients treated with salvage whole-prostate CA. In their report BRFS was 81% at 2 years and 71% at 5 years follow-up, with a longer BRFS in patients with a higher PSA decrease after treatment; in addition, a very low urinary incontinence rate was observed with only 2.7% of major adverse events. The results on BRFS were better than those obtained by Exterkate et al [47] and these authors also agreed with the positive prognostic value of PSA levels after treatment, but they suggested that the occurrence of serious complications such as urinary incontinence and fistula should not be underestimated.

Regarding IRE, in a prospective multicentric trial [48] including 37 intermediate- and high-risk patients’ Local control was achieved in 78% of patients and 16% developed metastatic disease with a median time of 8 months. In addition, 38% experienced complications of which 19% were grade 3 according to the Clavien-Dindo classification. An improved safety profile was demonstrated in the study by Geboers et al [49] with a complication rate of 12% (one rectal fistula and 8 urethral sloughings); in this series, 78% of patients obtained local control, the metastasis-free survival rate was of 91% with the same median time to metastases of [48] and a 5-year progression-free survival rate of 60%.

## Ablation in uterine fibroids

Uterine fibroids represent the most prevalent benign solid tumors found in the female genital tract. They manifest in approximately 20–25% of women of reproductive age [50]. Gynecologists and specialists in reproductive endocrinology and infertility commonly encounter these fibroids in patients who present with either singular or a combination of symptoms. These symptoms may include heavy menstrual bleeding, infertility, colicky dysmenorrhea [50, 51], and recurrent pregnancy loss.

The localization of uterine fibroids appears to play a crucial role in determining the frequency and severity of symptomatology. Traditional treatments involve abdominal myomectomy or hysterectomy, as these approaches have long been considered the standard surgical route for addressing symptomatic submucosal fibroids [52–59].

Historically, hysterectomy was routinely recommended for patients whose reproductive desires had been fulfilled, while abdominal myomectomy was considered the only viable option for young patients desiring pregnancy.

However, contemporary advancements have led to increased image-guided procedures and a growing array of pharmaceutical agents, each with value for appropriately selected and advised patients. Today, there is a trend toward minimally invasive treatment for uterine myomas [60].

The most used is uterine artery embolization (UAE), with adverse events related to possible postembolization syndrome, infection, pain, and potential damage to fertility [61].

Image-guided thermal ablation techniques, such as MWA, RFA, and image-guided HIFU ablation, have been used recently. The primary purpose of the ablation treatment is to improve the quality of life and alleviate the clinical symptoms.

Identifying the optimal approach requires the clinician to comprehensively understand the patient’s history, including her fertility desires.

Ultrasound-guided percutaneous MWA has emerged as a widely used method for treating symptomatic myomas and adenomyosis [62, 63].

This technique consists of percutaneous placement of a microwave antenna through the abdominal wall or vagina under US guidance into the tumor. The needle tip was located 5 mm from the distal end of the myoma to perform tumoral ablation. US guidance allows direct visualization of the needle and the ablation area during the procedure, increasing procedural safety; the ablative effect can be evaluated with ultrasound contrast at the end of the procedure [64].

In 2021, Liu et al conducted a meta-analysis of ten studies regarding 671 patients with symptomatic leiomyomas (average myoma diameter ranged between 4.9 cm and 7.2 cm) that underwent percutaneous or cervical MWA [65].

The results reported that the hemoglobin concentration increased significantly at the time of follow-up, as well as the quality of life (measured by uterine fibroid symptom and quality of life questionnaire scores). Furthermore, the reduction volume rate after the MWA treatment was 85% without major adverse events. The results also showed that the mean operation time was 34.48 min during MWA treatments, lower than HIFU therapy (with a mean operation time of 145.6 min) [65].

Liu et al conducted a comparative meta-analysis on the HIFU and surgery [66] for treating symptomatic uterine fibroids, analyzing ten studies with 4450 women (2483 of whom underwent HIFU and 1967 of whom underwent surgery). The results showed that the increase in quality of life was higher in the HIFU group compared with the surgery group. The duration of hospital stay and time to return to work were shorter in the HIFU group. The incidence of significant complications was lower in the HIFU group, with a statistically significant value. Instead, the differences in adverse events, symptom recurrence, re-intervention, and pregnancy outcomes were not statistically significant.

In 2020, Liu et al produced another comparative metanalysis of UAE and HIFU for treating symptomatic uterine myomas. A total of seven articles involving 4592 women were included in the metanalysis. The results showed that compared with the HIFU ablation group, the QoL scores at the follow-up time were higher in the UAE group. The women in the UAE group had a significantly lower reintervention rate and a significantly lower pregnancy rate than those undergoing HIFU ablation. No statistically significant adverse events were found [67].

In 2019, Bradley et al conducted a meta-analysis including 32 articles of 1283 patients treated with laparoscopic, transvaginal, or transcervical RFA fibroid ablation. The mean procedure time was 49 min, the time to discharge was 8.2 h, the time to normal activities was 5.2 days, and a decreased fibroid volume at 12 months was 66%. The results showed that RFA significantly reduces fibroid volume and improves fibroid-related quality of life [68].

Liu et al [65] reported a volume decrease of 85% after MWA therapy, Verpalen et al [69] reported a myoma shrinkage of 37.7% after HIFU treatment, and Bradley et al [68] reported a mean myoma volume decrease of 71% at > 12-month follow-up after RFA therapy. Furthermore, the ablation areas were more extensive for MWA treatment than for RFA up to 6 cm and 5 cm, respectively [70, 71].

From the above data, it can be deduced that compared with other thermal ablation techniques like HIFU and RFA, MWA is relatively simple, time-saving, and efficient in myoma volume reduction.

Other ablative techniques for treating symptomatic uterine fibroids include cryomyolysis [72] and laser ablation [73]. However, very limited evidence exists in the literature that reports directly on the efficacy of these techniques in treating fibroid-related bleeding symptoms.

Today, these techniques are still reserved for highly selected patients. All the observations were based on a limited sample size, requiring more in-depth research, particularly multicentric. Better comparative data are still needed. Further, randomized studies must provide sufficient and reliable data, especially on the re-intervention rate and pregnancy outcome.

## Final considerations

Ablative treatments are an established therapeutic reality in the oncological field. After the first work on the liver, this type of treatment spread has reached almost all districts. The application of ablative therapies in the management of genitourinary tract disease is constantly growing, leading to a huge scientific production, especially in the recent few years. Tissue characteristics and the need to achieve the greatest possible savings in vital structures adjacent to tumoral lesions are expanding the indications of CA and IRE whereas RFA and MWA confirm their role in this field (Table 1). The lack of randomized trials and trials comparing ablative techniques with other treatment options, including other ablative therapies, still makes unclear the potential role of ablative techniques in the management of genitourinary tract diseases.

**Table 1:** Recommendation for urogenital ablative treatments

Main results	Recommendations
RCC	
-Ablation is superior to LPN in terms of complications rate and residual renal function	1. Ablation techniques should be preferred to LPN in patients with peripheral RCC 2. Ablation alone should be avoided in lesions > 3 cm 3. Consider ablative re-treatment when LR occurs 4. Ablative technique (RFA, MWA, or CA) should be selected according to operator experience 5. Combined treatment (embolization + ablation) can be an option for lesions > 3 cm located in complex locations
-Ablation is equal to LPN in terms of LR and distant metastasis rate	
-Ablation is characterized by a possibility for retreatment after LR	
-LR is higher in central lesions > 3 cm	
-RFA, MWA, and CA provide comparable complication and survival rates	
-Combined treatment (embolization + ablation) increases the results in T1a tumors in challenging locations	
-Combined treatment (embolization + ablation) increases treatment coverage and reduces blood loss in lesions > 5 cm	
Adrenal disease	
- Ablation finds application in both benign and malign (primary and metastatic) adrenal disease	1. Ablation of adrenal disease should be considered in non-surgical patients as a second-line treatment 2. Alpha blockade should be considered prior to ablation in all patients, in order to reduce side-effects 3. Ablative technique (RFA, MWA, or CA) should be selected according to operator experience
-Ablation for benign disease effectively controls blood pressure, antihypertensive drug therapy, and hormone secretion	
-RFA, MWA, and CA show similar local control rates	
-Severe adverse events occur in 16% of patients after ablation	
-Intraoperative hypertension is the most frequent adverse event	
-Hypertensive crisis rate increases in the presence of normal adrenal tissue and when tumor diameter is smaller than < 4.5 cm	
-Ablation achieves good local control (81%) and OS (80%)	
-Ablation for aldosterone-producing adrenal adenoma can effectively control blood pressure, reduce the need for antihypertensive drugs, and normalize hormone secretion	
-Alpha blockade prior to ablation helps prevent hypertensive crises	
Prostate cancer	
-Ablative therapies find their role in the treatment of patients at intermediate risk in which the goals are the downstaging and the prevention of surgical/radiotherapies side effects	1. Ablation of prostate cancer should be considered in intermediate-risk patients and in recurrency as second-line treatment 2. Ablative technique (HIFU, CA, or IRE) should be selected according to operator experience
-HIFU, CA, and IRE are the most applied techniques	
-HIFU, CA, and IRE have similar oncological outcomes	
-Ablative therapies find application in recurrent prostate cancer	
-Ablation appears relatively safe with an acceptable level of side effects	
Fibroids	
-Ablation therapy improves the QoL and alleviates clinical symptoms	1. Surgery and UAE remain the first-line treatment for fibroids 2. MWA should be preferred to RFA for larger myomas 3. HIFU, CA, and Laser ablation have poor literature support to be applied in a clinical setting
-US-MWA is a simple, time-saving, and efficient emerging treatment option for achieving myoma volume reduction	
-HIFU appears to have a higher QoL score and lower incidence of significant complications as compared with surgery	
-HIFU appears to have a lower QoL score and higher reintervention rate as compared with UAE	
-MWA produces larger ablation areas than RFA	
-CA and laser ablation are less used	

*LPN* laparoscopic partial nephrectomy, *LR* local recurrence, *RFA* radiofrequency ablation, *MWA* microwave ablation, *CA* cryoablation, *RCC* renal cell carcinoma, *HIFU* high-intensity focused ultrasound, *US* ultrasound, *QoL* quality of life, *UEA* uterine artery embolization

## Abbreviation

ACTH: Adrenocorticotropic hormone
AMs: Adrenal metastases
AT: Ablation therapies
BRFS: Biochemical recurrence-free survival
CA: Cryoablation
CSS: Cancer-specific survival
HIFU: High-intensity focused ultrasound
IRE: Irreversible electroporation
LPN: Laparoscopic partial nephrectomy
LR: Local recurrence
MWA: Microwave ablation
OS: Overall survival
PN: Partial nephrectomy
RCC: Renal cell carcinoma
RFA: Radiofrequency ablation
TAE: Trans-arterial embolization
UAE: Uterine artery embolization
